# Pyogenic Flexor Tenosynovitis Caused by Shewanella putrefaciens

**DOI:** 10.7759/cureus.8113

**Published:** 2020-05-14

**Authors:** Anooj Patel, Mona Ascha, Ayesha Punjabi, Marco Swanson, Tobias C Long

**Affiliations:** 1 Plastic Surgery, Case Western Reserve University School of Medicine, Cleveland, USA; 2 Plastic Surgery, University Hospitals Cleveland Medical Center, Cleveland, USA; 3 Plastic Surgery, University Hospitals, Cleveland, USA

**Keywords:** dip: distal interphalangeal, flexor tenosynovitis, shewanella putrefaciens

## Abstract

Flexor tenosynovitis is a surgical emergency due to the risk of tendon necrosis which can lead to subsequent amputation. We report a case of flexor tenosynovitis with Shewanella putrefaciens as the implicated organism, though the patient’s mechanism of penetrating trauma did not involve a marine exposure. Shewanella are Gram negative bacilli associated with marine environments and have rarely been implicated in human disease. This patient presented with all four of Kanavel’s signs and required open surgical irrigation and debridement; he was found to have purulence but no flexor tendon necrosis. This case emphasizes the importance of considering marine organisms as putative for flexor tenosynovitis, even if marine exposure does not occur at the time of the penetrating trauma. It also emphasizes the need to obtain a thorough patient history, especially in cases of infection, to assess for all possible environmental exposures.

## Introduction

Flexor tenosynovitis is a closed-space infection of the flexor tendon sheath of the digit. The natural history of this condition is a direct result of the anatomy of the tendon sheath, a double-walled structure consisting of a visceral layer densely adherent to the tendon and a parietal layer adjacent to the pulley system [1]. Between these layers is synovial fluid, which is the first nutritional supply to the flexor tendon; the second is the vincular system. A closed system occurs because these visceral and parietal layers are connected at the proximal and distal extents of the tendon sheath: at the level of the metacarpal neck and just proximal to the distal interphalangeal (DIP) joint, respectively.

Upon inoculating the flexor tendon sheath, bacteria utilize the synovial fluid as a nutritional source and proliferate readily, as the poor vascular supply within the closed space limits the host defense. The closed nature of the tendon sheath also results in profound increases in volume and pressure, which in turn obstructs nutritional supply to the tendon via the vincular system [2]. The tendon is thus deprived of both of its nutritional sources, and imminently susceptible to necrosis, rupture, and contracture. For these reasons, flexor tenosynovitis represents a surgical emergency [3]. Typical microbiology in flexor tenosynovitis includes *Staphylococcus aureus*, beta-hemolytic Streptococcus, or *Pasturella multicoda* in cases involving animal bites; Mycobacterium and Vibrio species are most commonly associated with water exposure [3-4].

We present the unusual case of a *Shewanella putrefaciens* flexor tenosynovitis resulting from penetrating trauma via a drill bit in a patient who owns a puffer fish and maintains a fish tank. To our knowledge, *Shewanella* species (*Shewanella algae*) has only been implicated in flexor tenosynovitis once prior in the literature, and this was associated with a fish hook injury [4].

## Case presentation

A 38-year-old right-handed male presented to the ER for left index finger pain and edema. Four days prior, the patient sustained a penetrating injury to the left index finger volar DIP crease via a new, clean drill bit which he was using at home. The patient did not seek medical care at that time, washed the wound with peroxide, applied bacitracin, and bandaged himself. Over the next few days, he experienced worsening pain and swelling. The day before presentation, the patient sustained further trauma to the finger when it caught in a door hinge. Hand surgery was called due to concern for flexor tenosynovitis. The patient endorsed no symptoms other than finger pain. Significant history for the presenting complaint included a prior left thumb dislocation and bilateral carpal tunnel releases.

Past medical history included a history of military service causing traumatic brain injury, depression, anxiety, migraines, fibromyalgia, and arthritis. Past surgical history included a left neck melanoma resection. The patient lives on a farm and works as a semi-retired mechanic; he has sustained many lacerations to his hands bilaterally due to his work, and works in the dirt frequently. The patient takes medications for depression and anxiety and has no known allergies. The patient is a former smoker and occasional drinker, and denies illicit drug use. The patient uses well water at home, denies any recent water exposure including rivers, ponds, or oceans, however, did admit to having a fish tank and a puffer fish at home.

**Figure 1:**
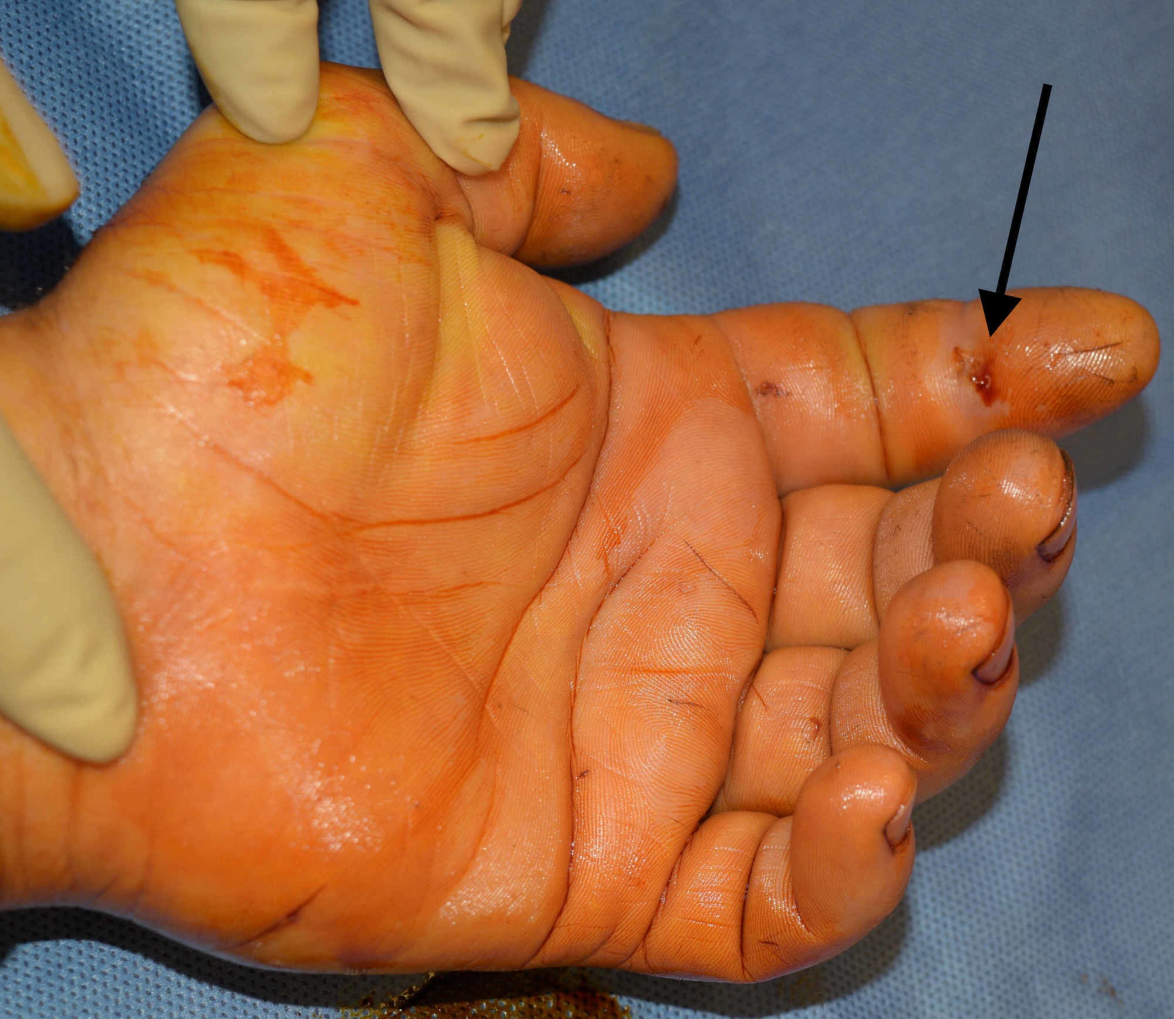
Preoperative photograph of left index finger demonstrating site of penetrating injury. Black arrow points to the exact location of the penetrating injury.

On physical exam, the patient was afebrile and in no distress. There was a small puncture wound on the volar DIP crease of the left index finger (Figure 1). No pus or drainage could be expressed from the wound. All four Kanavel signs were present: 1) pain on passive extension, 2) the digit was held in a flexed posture, 3) fusiform swelling of the digit, and 4) tenderness to palpation from the A1 pulley to the DIP crease. Range of motion was limited secondary to pain. Both of the patient’s hands were dirty with many scattered small scars from prior injuries from work. The remainder of the hand exam was unremarkable. Basic metabolic panel and complete blood count were within normal limits. Left hand X-ray demonstrated soft tissue swelling of the left index finger without evidence of acute fracture or dislocation.

**Figure 2:**
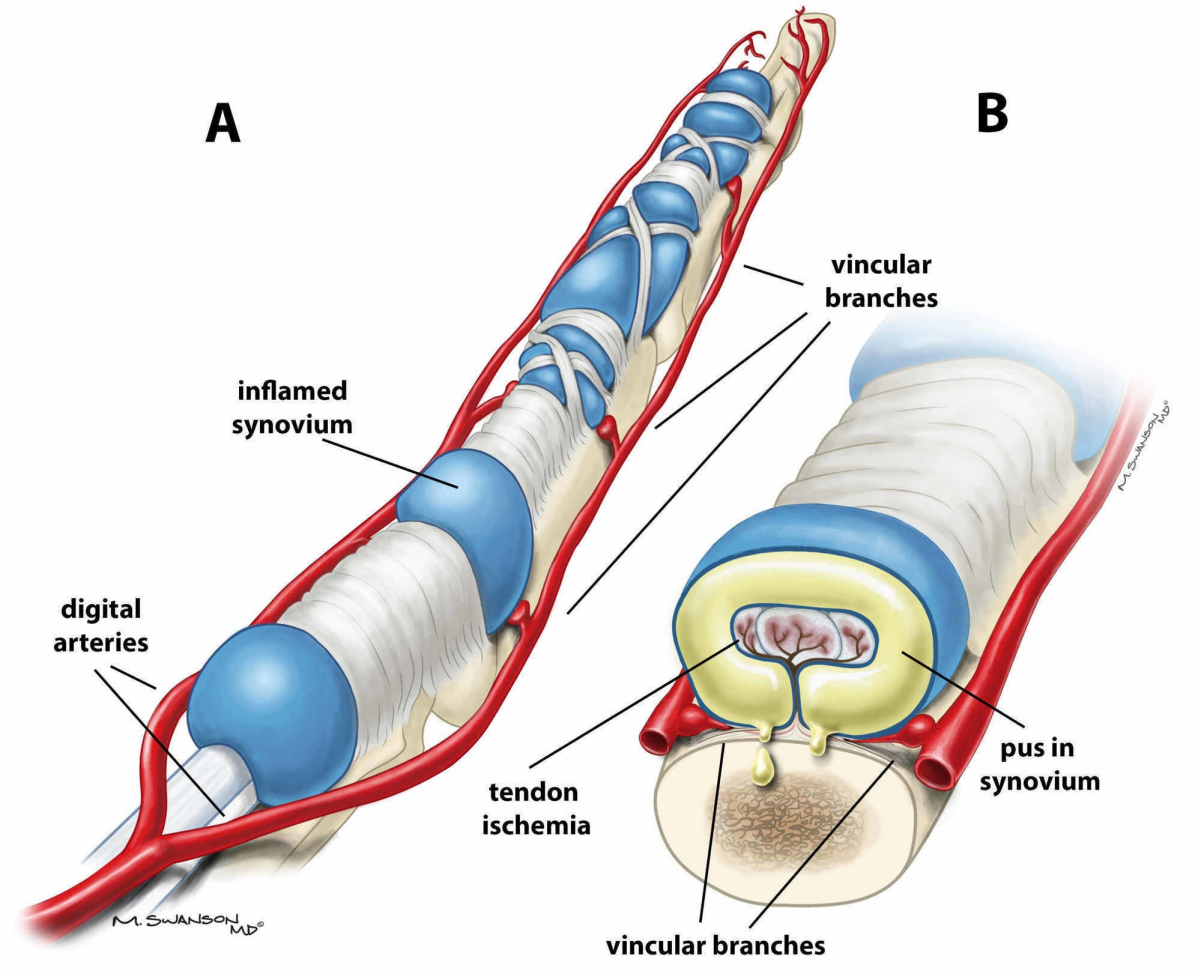
A) Anatomy of flexor tendon sheath with swollen sheath due to pyogenic flexor tenosynovitis. B) Cross section of flexor tendon sheath demonstrating pus in the sheath obstructing blood flow from the vincular branches.

**Figure 3:**
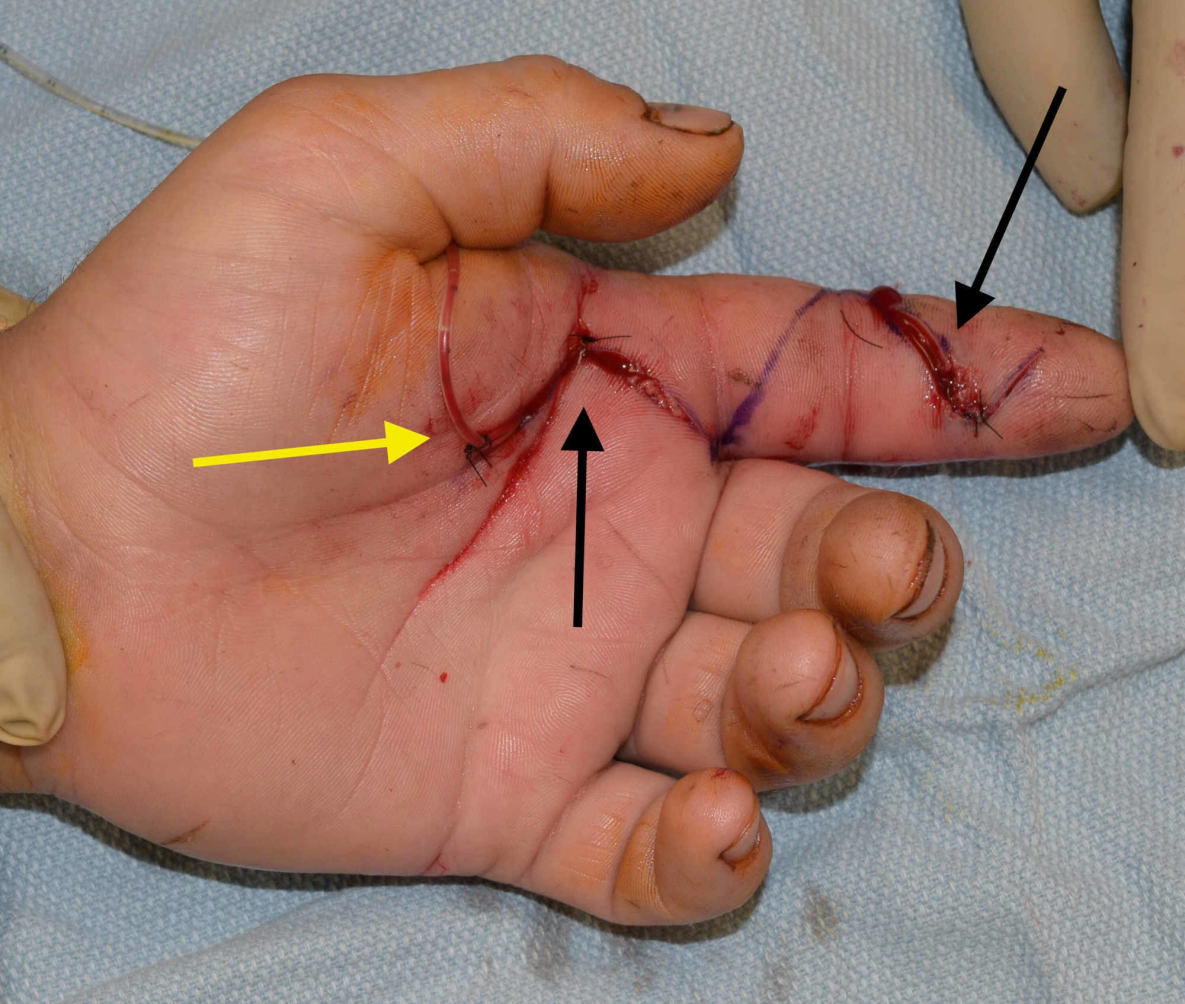
Postoperative photograph of left index finger demonstrating distal and proximal Bruner’s incisions with irrigation catheter placed in flexor tendon sheath. Black arrows point to the distal and proximal Bruner's incisions. Yellow arrow points to catheter insertion into flexor tendon sheath.

The patient was given a tetanus booster, started on empiric vancomycin and piperacillin/tazobactam, and taken to the operating room emergently. The flexor tendon sheath was approached via a Bruner incision at the volar distal phalanx. Gross purulence was expressed from the distal end of the flexor tendon sheath, confirming the diagnosis of flexor tenosynovitis. A counter-incision in the palm enabled evaluation of the A1 pulley; gross purulence was also expressed at the proximal end of the A1 pulley, and it was subsequently released. Cultures were sent from both proximal and distal sites. The flexor tendon sheath was copiously irrigated and a pediatric feeding tube was placed in the tendon sheath and continuously irrigated with 60 cc of 2% lidocaine in a liter of normal saline for 48 h. Please refer to Figure 2A,B for the relevant anatomy in pyogenic flexor tenosynovitis. The skin flaps were loosely tacked down with 3-0 nylon suture (Figure 3). 

 

Postoperatively, the patient’s symptoms improved. On postoperative day one, distal tendon sheath cultures grew methicillin-sensitive *S. aureus*, *Klebsiella oxytoca*, and *S. putrefaciens*, the latter of which was thought to be related to fish tank and puffer fish exposure (Table 1). Proximal tendon sheath cultures grew *S. aureus* (Table 2). On postoperative day two, the irrigation catheter and dressing were removed, the infectious disease team was consulted, and the occupational therapy was initiated. On postoperative day three, the patient was discharged home with trimethoprim/sulfamethoxazole double strength 800-160 mg oral tab BID for 14 days to treat *S. aureus* and *K. oxytoca*, and ciprofloxacin 500 mg BID for 14 days to treat the *S. putrefaciens*. Further sensitivity testing for *S. putrefaciens* revealed sensitivity to trimethoprim/sulfamethoxazole, and the patient was switched to monotherapy to treat all three organisms.

**Table 1:** Cultures and sensitivities from distal tendon sheath.

Antibiotic	Shewanella putrefaciens	Klebsiella oxytoca	Staphylococcus aureus
Gentamycin	Sensitive	Sensitive	Sensitive
Cefepime	Sensitive		
Ceftazidime	Sensitive		
Ciprofloxacin	Sensitive	Sensitive	Sensitive
Tobramycin	Sensitive		
Piperacillin/Tazobactam	Sensitive	Sensitive	
Trimethoprim/Sulfamethoxazole	Sensitive	Sensitive	Sensitive
Ampicillin		Resistant	Resistant
Amoxicillin/Clavulanate		Sensitive	
Cefazolin		Sensitive	
Levofloxacin		Sensitive	Sensitive
Clindamycin			Sensitive
Erythromycin			Sensitive
Oxacillin			Sensitive
Penicillin			Resistant
Tetracycline			Sensitive
Vancomycin			Sensitive

**Table 2:** Cultures and sensitivities from proximal tendon sheath.

Antibiotic	Staphylococcus aureus
Ampicillin	Resistant
Clindamycin	Sensitive
Ciprofloxacin	Sensitive
Erythromycin	Sensitive
Gentamicin	Sensitive
Levofloxacin	Sensitive
Oxacillin	Sensitive
Penicillin	Resistant
Trimethoprim/Sulfamethoxazole	Sensitive
Tetracycline	Sensitive
Vancomycin	Sensitive

On the day of discharge, the patient was afebrile, with significant improvement of finger swelling and pain. Range of motion was improved but still limited due to stiffness. The patient was advised to follow up in clinic in three weeks. At the follow up appointment, the patient’s incisions were healed, the antibiotic course was completed, and finger flexion had almost fully returned. He will continue occupational therapy for range of motion.

## Discussion

*Shewanella* is a nonfermenting Gram negative rod naturally present in marine water and soil; it was initially isolated at the beginning of the twentieth century from putrefied butter and has thereafter also been isolated from fish, sewage, and carcasses. Out of the thirty species of *Shewanella*, only two (*S. putrefaciens* and *S. algae*) have been implicated in human diseases, including bacteremia, cellulitis, necrotizing fasciitis, otitis media, open lower extremity fractures, spondylodiscitis, and pneumonia [5-11]. Though flexor tenosynovitis secondary to *S. algae* has been described, and *S. putrefaciens* has been implicated in soft tissue infections of the hand [4,12-13], to our knowledge this is the first reported case of *S. putrefaciens* flexor tenosynovitis caused by a penetrating injury. 

Of note, *S. algae* flexor tenosynovitis presents with a much more rapid course when compared to *Mycobacterium marinum*, the more common pathogen in flexor tenosynovitis associated with marine exposures [4]. The authors of this case report delineate the severity of flexor tenosynovitis at presentation via Michon’s progression [14]:

Stage I: serous exudative fluid with viable tendon

Stage II: purulent fluid with viable tendon

Stage III: septic necrosis of the tendon and pulleys

The reported case of *S. algae* flexor tenosynovitis presented at stage III, is shown by comparison in Table 3, while *M. marinum* has been noted to present at stage I [4, 15]. Our patient presented at Michon stage II, suggesting that *S. putrefaciens* may be intermediate in virulence between *S. algae* and *M. marinum*. This is consistent with the suggestion that *S. algae* is the most virulent species of the *Shewanella* genus [16]. *M. marinum*, meanwhile, follows a more indolent clinical course, with an incubation period of several weeks.

**Table 3:** Classification comparison between our case: Shewanella putrefaciens, and an existing case: Shewanella algae. Table includes data adapted from a case report on *Shewanella algae* [4].

	Shewanella putrefaciens case	Shewanella algae case
Kanavel signs		
Fusiform swelling	Yes	Yes
Finger in flexion	Yes	Yes
Pain on passive extension	Yes	Yes
Flexor sheath tenderness	Yes	Yes
Fever	No	Yes
Elevated white blood cell count	No	Yes
Risk factors associated with poor outcome		
Age > 43 years	No	No
Presence of diabetes mellitus, peripheral vascular disease, or renal failure	No	No
Subcutaneous purulence	Yes	Yes
Digital ischemia	No	Yes
Polymicrobial infection	Yes	Yes
Michon classification		
Stage I	No	No
Stage II	Yes	No
Stage III	No	Yes

The classification system proposed by Pang et al delineates risk for poor outcomes in flexor tenosynovitis, particularly digit amputation [17]. The following factors were found to be significant:

1. age greater than 43 years

2. presence of diabetes mellitus

3. peripheral vascular disease or renal failure

4. subcutaneous purulence

5. digital ischemia

6. polymicrobial infection

The fourth and sixth risk factors were applicable to our patient.

*Shewanella* has demonstrated resistance to penicillin, but susceptibility to ampicillin-sulbactam, piperacillin-tazobactam (which our patient received), cephalosporins, aminoglycosides, and fluoroquinolones [7]. Fortunately for our patient, all three organisms implicated in his infection were sensitive to trimethoprim-sulfamethoxazole.

Unique aspects of our case include *S. putrefaciens* as the putative organism in flexor tenosynovitis, and the disconnect between the mechanism of penetrating injury to the flexor surface of the finger and the marine exposure. Our patient’s penetrating injury was with a clean drill bit, and likely exposure to *Shewanella* was from caring for his puffer fish. It is unclear whether *Shewanella* had previously colonized the skin flora exposed to the penetrating injury or if the exposure was afterwards. This emphasizes that marine organisms including *Shewanella* should be considered for any marine exposure in flexor tenosynovitis, even if separates from the penetrating trauma. In addition, a thorough history should be obtained at the time of injury.

## Conclusions

Flexor tenosynovitis is a closed-space infection of the flexor tendon that requires urgent diagnosis and treatment with both surgery and antibiotics. The high risk of tendon necrosis can lead to amputation of the digit if not treated correctly. Organisms that have been commonly known to inoculate the sheath after a penetrating injury in various settings include *S. aureus*, beta-hemolytic Streptococcus, *P. multicoda*, Mycobacterium, and the Vibrio species. Rarer species may also be involved, and thus a detailed patient history is important to start appropriate treatment. This case highlights the importance of broadening the possible infectious microbiology to include *Shewanella*, when patients have a history of exposure to marine organisms, even if the exposure is not at the time of the penetrating injury. *Shewanella algae* may be more virulent in comparison to *S. putrefaciens*. 
